# Influence of Acidic Environment on the Hardness, Surface Roughness and Wear Ability of CAD/CAM Resin-Matrix Ceramics

**DOI:** 10.3390/ma15176146

**Published:** 2022-09-05

**Authors:** Wejdan S. Alghamdi, Nawaf Labban, Ahmed Maawadh, Hussain D. Alsayed, Huda Alshehri, Ali Alrahlah, Sarah M. Alnafaiy

**Affiliations:** 1 Department of Prosthetic Dental Sciences, College of Dentistry, King Saud University, Riyadh 11545, Saudi Arabia; 2 Department of Restorative Dental Sciences, College of Dentistry, King Saud University, Riyadh 11545, Saudi Arabia; 3 Department of Restorative Dental Sciences, Engineer Abdullah Bugshan Research Chair for Dental and Oral Rehabilitation, College of Dentistry, King Saud University, Riyadh 11545, Saudi Arabia

**Keywords:** CAD/CAM technologies, microhardness, resin ceramics, surface roughness, wear

## Abstract

This study aimed to measure the effect of storage environment on the hardness, surface roughness and wear ability of CAD/CAM resin-matrix ceramics. A total of 200 rectangular-shaped specimens were obtained by sectioning 5 CAD/CAM blocks; Crystal Ultra (CU), Vita Enamic (VE), Lava Ultimate (LU), Cerasmart (CS) and Vita blocks Mark II (MII). Microhardness and surface roughness were measured at baseline and after 7 days of immersion either in saliva or cola (n = 10). The wear ability of the CAD/CAM materials against steatite-ceramics antagonist was determined using a chewing simulator. The data were statistically analyzed using factorial ANOVA followed by post hoc Bonferroni multiple comparison tests (*p* < 0.05). The independent factors significantly influenced the microhardness and surface roughness (*p* < 0.05). The highest VHN was observed in MII at baseline (586.97 ± 13.95), while CU showed the lowest VHN after 7 days of immersion in cola (68.3 ± 1.89). On the contrary, the highest Ra was observed after 120,000 chewing cycles for the VE specimens (1.09 ± 0.43 µm) immersed in cola, while LU showed the lowest Ra at baseline (0.07 ± 0.01 µm). The highest % mass loss of the antagonist was observed with MII immersed in cola (1.801%), while CS demonstrated the lowest % mass loss of 0.004% and 0.007% in AS and cola, respectively. This study confirms that the surface properties of tested CAD/CAM materials are susceptible to degradation in an acidic environment except for hardness and wear of CS material.

## 1. Introduction

Recently, there have been paradigm shifts towards using resin-ceramic indirect restorations as an alternative to all-ceramic restorations. This suggests that this new material category has the advantage over all-ceramic material in terms of chipping resistance, reparability and wear resistance, as well as a low brittleness index [1]. The elastic modulus of resin-ceramic materials is close to dentin and therefore a uniform stress distribution at the dentin-restoration interface is favorable [2], providing durable restorations under chewing forces [3,4].

Among the available restoration systems, computer-aided design and computer-aided manufacturing (CAD/CAM) allows for rapid production of tooth-colored restorations both for resin ceramic and all-ceramic materials [5]. Industries produce CAD/CAM composite resin blocks using defined parameters at high pressure and temperature to obtain the desired qualities at the microstructure level [6]. The investigators claim that the production and application of restorations prepared with CAD/CAM technology systems provide better performances than restorations performed with conventional laboratory procedures in terms of esthetics, clinical life and marginal precision [7,8,9].

One of the important factors to consider when selecting a restorative material is its mechanical properties. Restorative materials must be strong enough to withstand the stresses of mastication because they are used to replace missing tooth structures [8,10]. Hardness is a good predictor of the mechanical qualities of dental materials and is defined as the materials resistance to permanent indentation or penetration. Ceramic material hardness can have an impact on the material’s machinability, polishability and wear resistance, and is frequently influenced by ageing, water absorption and surface reactions [11]. Different hardness tests, including the Knoops, Vickers and Martens can be used to measure hardness. However, researchers have frequently used Vickers microhardness tests to determine the hardness of dental materials [12]. Surface roughness of a dental restorative material is also deemed a vital parameter for the longevity of restoration [13]. Rough surfaces can retain plaque and stains, making it difficult to maintain good oral hygiene. This is critical in dentistry because surface behavior is linked to use and scratch, implying that resistance is linked to clinical long-term efficacy.

In today’s world, wear of restoration has become a common and serious problem. Restorative materials are exposed to a complex environment in the oral cavity, where they undergo severe chemical and physical stresses due to temperature changes, functional and parafunctional loads and chemicals from food and drinks [14,15], especially the erosive potential of acidic drinks [16]. To provide long-term stability, dental materials should demonstrate good wear resistance [17]. There are different CAD/CAM materials available to clinicians for fabricating dental restorations and these materials are expected to demonstrate high resistance to wear and indentation, owing to the industrial polymerization process [8]. Previous studies evaluating the new resin-ceramic materials have focused mainly on properties related to flexural strength or flexural modulus [18,19], flexural strength or hardness [20] or the color stability [21]. The data regarding the surface properties and wear of CAD/CAM resin-matrix ceramic restorative materials are scarce. The evaluation of these new generations of CAD/CAM resin-matrix ceramic materials immersed in acidic beverage could provide useful information regarding the materials’ tendency to hardness, roughness and wear in acidic conditions.

Therefore, this laboratory study aimed to evaluate and compare the hardness, surface roughness and wear ability of CAD/CAM resin-matrix ceramic restorative materials following exposure to an acidic environment and simulated chewing. The first null hypothesis tested for this study was that no significant difference would be detected in the microhardness of the tested CAD/CAM ceramic materials. The second null hypothesis was that no significant difference would be detected in the surface roughness of the tested CAD/CAM ceramic materials. The third null hypothesis tested was that no significant difference would be detected in the wear ability of the tested CAD/CAM ceramic materials against the antagonist.

## 2. Materials and Methods

Four CAD/CAM resin-matrix ceramic materials—Crystal Ultra (CU) (Digital Dental, Scottsdale, AZ, USA), Vita Enamic (VE) (VITA Zahnfabrik, Bad Säckingen, Germany), Lava Ultimate (LU) (3M ESPE, Irvine, CA, USA) and Cerasmart (CS) (GC Corporation, Tokyo, Japan), and one feldspathic ceramic material, Vitablocs Mark II (MII) (VITA Zahnfabrik, Bad Säckingen, Germany)—were evaluated.

### 2.1. Specimen Preparation and Distribution

**Figure 1:**
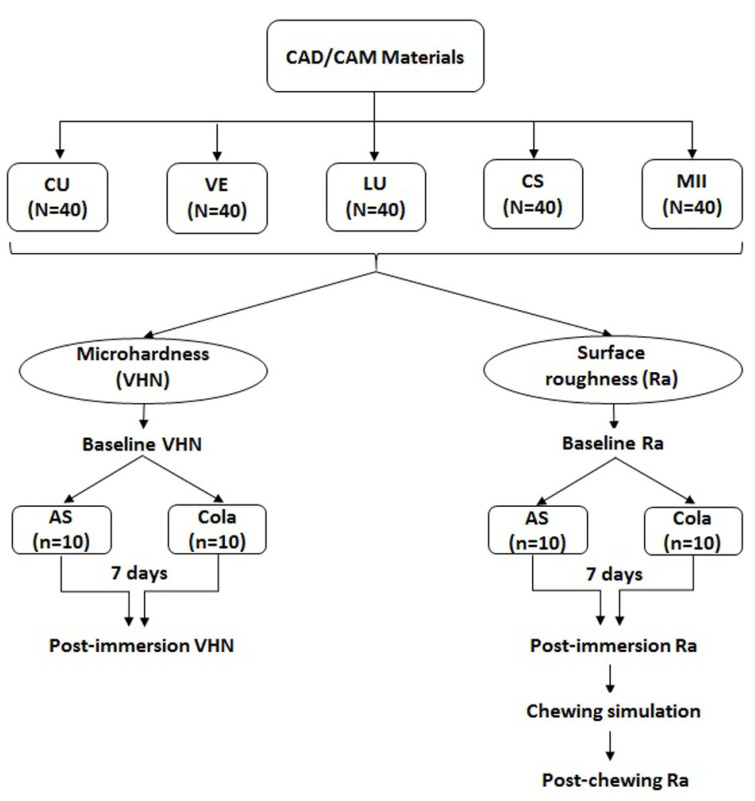
Specimen distribution and test set-up.

A total of 200 rectangular specimens were prepared using the selected CAD/CAM blocks. One hundred specimens were used for hardness measurement and the remaining 100 specimens were used for surface roughness and antagonist wear measurements. Figure 1 presents the specimen distribution and study set-up. A milling device (Ceramill Motion 2, Amann Girrbach AG, Koblach, Austria) was used to section the blocks to the desired dimensions (10 × 4 × 3 mm^3^). Next, the individual specimen was embedded in an epoxy resin using a silicon mold to produce cylindrical specimens measuring 25 mm in diameter and 10 mm in height. The specimens were then polished using a polishing machine (LaboPol-25, Struers, Copenhagen, Denmark) with sequential use of silicon carbide papers (400, 600, 800, 1000 and 1200 grit sizes) at 300 rpm under a water coolant. Following polishing, the specimens were cleaned with distilled water in an ultrasonic bath (Quantrex 90 WT, L & R Manufacturing, Inc., Kearny, NJ, USA) for 10 min and air-dried for 40 s [20] and stored in an incubator (JSGI-150T, JS Research Inc., Gongju, Korea) at 37 °C for 24 h.

### 2.2. Hardness (VHN)

The hardness of the specimens was evaluated using a microhardness tester (INNOVATEST Europe BV, Maastricht, The Netherlands) equipped with a Vickers diamond indenter. Three indentations were placed on each specimen at a distance of at least 0.5 mm and the mean value was considered as the reading for that particular specimen. During indentation, a load of 10 N was applied for 15 s [7]. The Vickers hardness number (VHN) was calculated using the below equation [20]:

H = 1.854 × F/d^2,^

where H = Vickers hardness number, F = load (in N) and d = area of the indentation diagonal length (in mm^2^).

### 2.3. Surface Roughness (Ra)

A 3D non-contact optical profilometer (Contour GT-X, Bruker, CA, USA) was used for surface roughness measurements. The specimen was mounted on the automated x-y stage and scanned using white light interferometry without making contact with the specimen surface. The scanning parameters included magnification of 5× with a nano-lens, 1 × 1 mm^2^ field of view, 1× scan speed and 0.1 mm/s stage speed. A Vision 64 (ver. 5.30, Bruker, CA, USA) proprietary software was used to control the x-y stage movements, precise control and accurate measurement of the selected specimen surface area. The specimen was scanned at three equidistant locations, and the mean of the three readings corresponded to the specimen’s surface roughness.

### 2.4. Immersion Process

Following baseline measurements, the respective specimens from the hardness and roughness categories were randomly allocated into two groups (n = 10) according to the immersion process. The specimens were either immersed in artificial saliva (AS) (pH 7.0) or acidic cola drink (pH 2.35). Artificial saliva was prepared at the College of Pharmacy in King Saud University per previous study [22]. The specimens were suspended inside a container containing 50 mL of either of the solutions using dental floss [7]. The container was stored in an incubator (Thermo Fischer Scientific, Waltham, MA, USA) at 37 °C for 7 days and the solutions were changed daily. Following the conclusion of the immersion process, the specimens were removed from the container and cleaned with a soft toothbrush under running water to remove any remnants from the surface.

### 2.5. Surface Wear

The surface wear ability of the tested CAD/CAM materials against the steatite-ceramic antagonist (Ø 6 mm, SD Mechatronik GmbH, Feldkirchen-Westerham, Germany) was evaluated using a computer-controlled chewing simulator (Proto-tech, Portland, OR, USA). The antagonist’s baseline mass in grams was determined using an analytic electronic balance (Precisa EP 225 SM-DR, Precisa Gravimetrics AG, Zurich, Switzerland). The specimens were positioned and secured in the lower sample holder of the device and the antagonist was affixed to a metal stylus in the upper sample holder. A chewing force of 49 N and 120,000 chewing cycles at a frequency of 1.6 Hz were applied to simulate approximately 6 months of the clinical life in accordance with previous studies [21,23,24]. After simulated chewing, the steatite–ceramic antagonist placed against each CAD/CAM specimen were removed from the sample holder, rinsed in running water and then dried and weighed to obtain the final mass. The percent mass loss of the antagonist against the CAD/CAM materials was calculated as below:

$$\mathrm{Percent}\;\mathrm{Change}\;\mathrm{in}\;\mathrm{Mass}=\frac{Mass\;difference}{Baseline\;mass}.$$

### 2.6. Statistical Analysis

The data were analyzed using Statistical Package for the Social Sciences (IBM SPSS Inc., v.22, Chicago, IL, USA) software. The normality of the data was confirmed with the Kolmogorov–Smirnov test. The descriptive statistics were obtained to compare the means of the study groups, while inferential statistics (three-way ANOVA) were calculated to observe any statistical variation between the study groups. Post hoc Bonferroni test was used for multiple comparisons. The weight loss of the antagonist was evaluated in percentage. A *p* value ≤ 0.05 was considered statistically significant for all the tests.

## 3. Results

Three-way ANOVA demonstrated that the independent factors (i.e., materials, storage environment and treatment time) significantly influenced the microhardness (*p* < 0.05). The interactive effect of independent factors and the cumulative effect of all the three independent factors also had a significant effect on the microhardness (*p* < 0.05) (Table 1).

**Figure 2:**
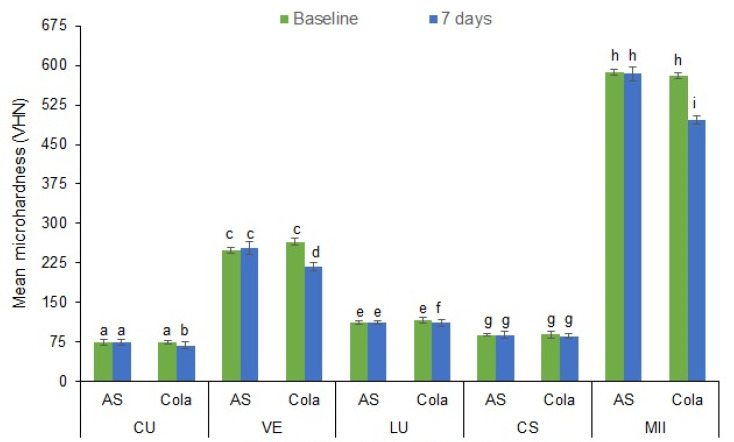
Mean microhardness (VHN) values of the CAD/CAM materials immersed in AS and cola at baseline and after 7 days immersion in either AS or cola. Bars indicate standard deviation. Post-hoc interpretation: Same lower-case alphabet shows the non-significant difference between the CAD/CAM material groups.

**Figure 3:**
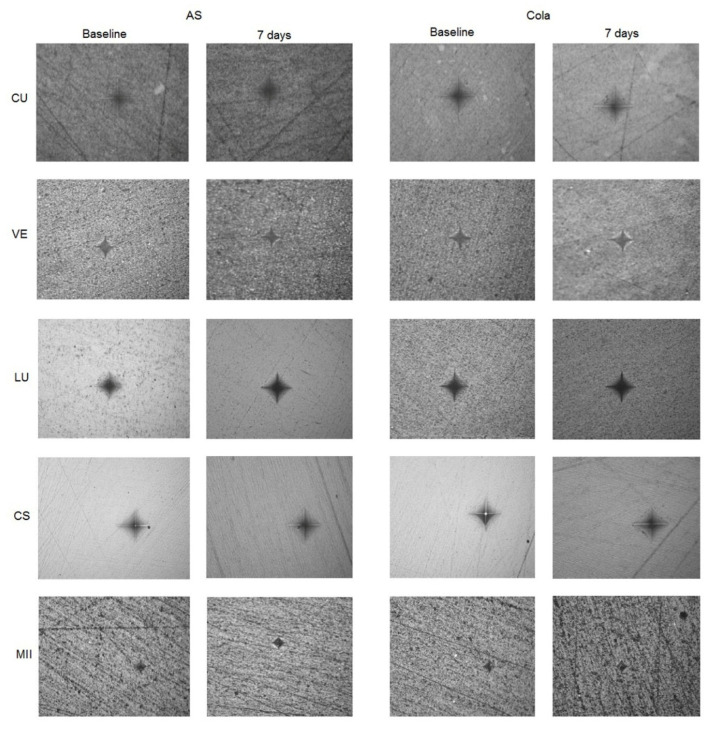
Vickers indentation images (×40) of the CAD/CAM specimens at baseline and after immersion in either AS or Cola.

Figure 2 presents the mean and standard deviation of the microhardness (VHN) values of the study groups at baseline and after 7 days of immersion in AS or cola. The highest VHN was observed in MII at baseline (586.97 ± 13.95), while CU showed the lowest VHN after 7 days of immersion in cola (68.3 ± 1.89). The immersion of specimens in AS demonstrated slightly decreased VHN but was non-significant (*p* < 0.05), while immersion in cola showed a significantly reduced VHN for all the groups except CS, where it was statistically insignificant (*p* > 0.05). Figure 3 presents the Vickers indentation images of the specimens at baseline and after 7 days of immersion either in AS or Cola.

Table 2 shows the outcome of three-way ANOVA demonstrating the influence of independent factors (i.e., materials, storage environment and treatment time) on surface roughness. Both materials and storage environment had a significant effect on the surface roughness (*p* < 0.05). However, treatment time had an insignificant effect (*p* = 0.828). The interactive effect of materials with storage environment and treatment time was found to be statistically significant (*p* < 0.05). On the contrary, the interactive effect of the storage environment with treatment time and the cumulative effect of all the three independent factors had no significant effect on the surface roughness (*p* = 0.646 and *p* = 0.661, respectively).

**Figure 4:**
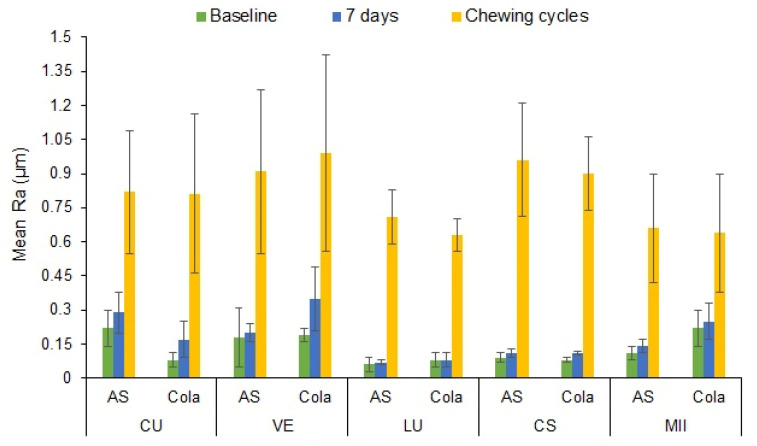
Mean surface roughness (Ra) of the CAD/CAM materials immersed in AS and cola at baseline and 7 days of immersion in either AS or cola, and after the chewing cycles. Bars indicate standard deviation.

Figure 4 presents the mean and standard deviation of the surface roughness (Ra) values of the study groups immersed at baseline, 7 days immersion in either AS or cola, and after 120,000 chewing cycles. The highest Ra was observed after 120,000 chewing cycles for the VE specimens (1.09 ± 0.43 µm) immersed in cola, while LU showed the lowest Ra at baseline (0.07 ± 0.01 µm). The post hoc Bonferroni multiple comparison tests suggested insignificant differences in Ra between the groups at baseline and after 7 days immersion (*p* = 0.195). However, after 120,000 chewing cycles, the groups showed statistical differences in Ra compared to baseline and 7 days treatment time (*p* = 0.000) for both storage environments.

**Figure 5:**
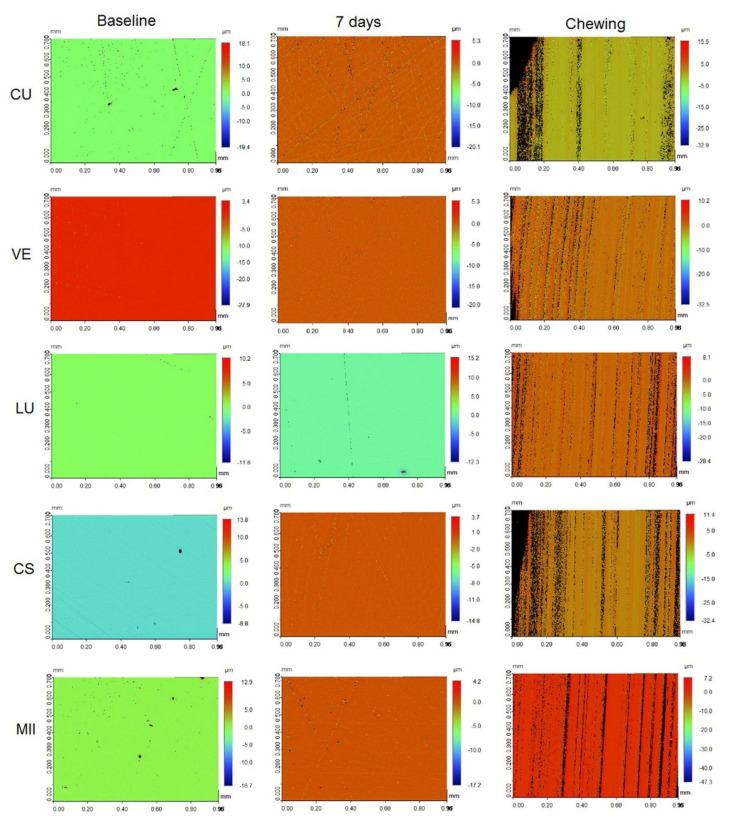
Profilometer images of the CAD/CAM specimens immersed in artificial saliva at different measurement intervals.

**Figure 6:**
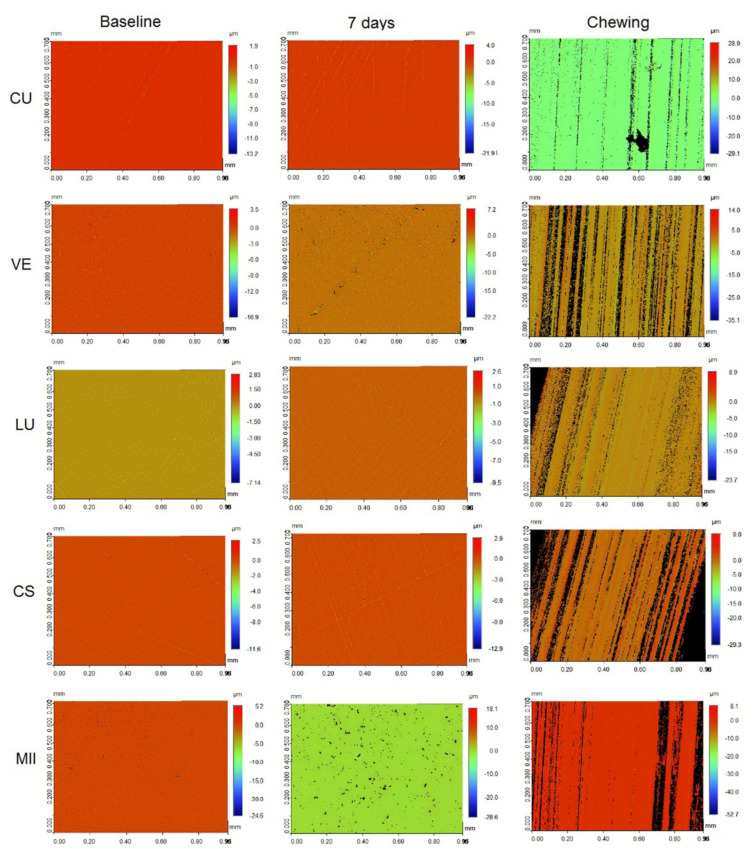
Profilometer images of the CAD/CAM specimens immersed in cola at different measurement intervals.

Figure 5 and Figure 6 presents the profilometer images of the specimens at different measurement intervals stored in either AS or cola, respectively. Irrespective of the immersion solutions, the specimens following the chewing simulation showed significant changes in roughness profile compared to the baseline and the roughness measurements after 7 days.

Table 3 presents the percent mass loss of the antagonist (6 mm steatite-ceramic ball) subjected to 120,000 chewing cycles against the tested CAD/CAM materials. The findings suggest the highest % mass loss of the antagonist against MII immersed in cola (1.801%) followed by MII immersed in AS (1.231%). In contrast, CS material demonstrated the lowest % mass loss of the antagonist, 0.004% and 0.007%, in AS and cola, respectively. Among the resin-matrix ceramic materials, VE immersed in both AS (0.022%) and cola (0.024%) showed increased wear of the antagonist.

## 4. Discussion

The present laboratory study suggests significant variations in the microhardness values between the study groups concerning storage environment and treatment time. Thus, the first null hypothesis is rejected. Characterization through hardness is an important step to evaluate the clinical life of a material [22]. In the current study, MII showed the highest microhardness values compared to other groups, which could be related to the peculiar characteristics of ceramic material, as this material has smaller and more homogenous crystal sizes with enhanced interlocking between them [23]. A significantly lower microhardness was observed among the study groups immersed in cola compared to those specimens immersed in AS. Our findings are in line with the previous studies [8,23] that advocate the deleterious effect of an acidic environment on the microhardness of the glass-ceramic material. Although the ceramic material is considered chemically inert, MII showed a deleterious effect after immersion in cola for 7 days. The reason could be the acidic and erosive nature of the low pH solution [24]. The continuous immersion of a ceramic material in a low pH solution dissolves the ceramic material and elementary components such as silica, potassium and aluminum released by the glassy phase [8]. Our baseline findings of MII hardness values are in line with previous studies [20,25].

Among the resin-matrix ceramic materials, VE showed the highest microhardness. The reason could be due to its composition, which contains a polymer-infiltrated feldspar ceramic network enriched with Al_2_O_3_ particles (i.e., 86 wt.%) and SiO_2_, Na_2_O and K_2_O particles in a urethane dimethacrylate (UDMA) polymer matrix [26]. The obtained hardness value of VE material is per the previous papers [27,28]. Similarly, LU is a resin-based ceramic nanofill composite material. The reported microhardness of LU in previous studies [29,30] closely matches the findings of our research. However, the marked difference in the hardness values of VE and LU can be attributed to the composition of the two materials.

It is noteworthy that the microhardness of CS was not affected by the storage environment and the treatment time. This hybrid ceramic material has a flexible nano-ceramic matrix structure, as claimed by the manufacturer. However, very few laboratory studies are available, and those findings correlate with the microhardness values of the present study [20,30]. CU is a recently introduced resin matrix-ceramic with a higher polymer/ceramic ratio. There is no published data to compare the findings of microhardness of CU in an acidic environment. However, slightly lower hardness of the CU compared to the CS among baseline groups is in accordance with a previous study [20]. Although, the previous study suggests the deleterious effect of acidic drinks on the microhardness [8] of CS, the present findings demonstrate an insignificant decrease in microhardness values, irrespective of the storage media and treatment time. The reason might be the flexible nature of this new material and presumably because of the chemical stability of the polymeric content. Furthermore, the comparison with previous findings should be done carefully due to the differences in the materials and methodology applied.

Regarding surface roughness, a significant difference within and between the study groups were observed. Hence, our second null hypothesis was also rejected. Surface roughness is an important parameter and can promote plaque accumulation and initiation of secondary caries [13]. The higher surface roughness of VE compared to LU could be attributed to micro-structured SiO_2_ fillers in VE and the nano-structured SiO_2_ and ZrO_2_ fillers in LU [31]. In the current study, the slight increase in surface roughness of the specimens after immersion in storage media among resin-matrix ceramic groups might be attributed to the presence of UDMA in resin-matrix ceramic materials. UDMA resin has polarity and affinity to water, thereby softening the polymer [32]. In contrast, an unnoticeable increased roughness of MII, irrespective of the immersion environment used, suggests the chemical stability and inertness of the glass-ceramic material [33].

We used a steatite-ceramic ball at a load of 49 N to standardize and represent physiological occlusal forces in a normal individual during chewing simulation. Significant differences in % mass loss of antagonists were observed among the groups, thereby suggesting the rejection of the third null hypothesis. CS showed the least wear of the antagonist followed by LU, while MII demonstrated significantly excessive wear of the antagonist. Among the resin-matrix ceramic groups, VE demonstrated increased wear of the antagonist (0.022% and 0.024% in saliva and cola, respectively). This might be due to the presence of hard micro-sized fillers (ca. 86 wt.%) [26]. The less wear of the antagonist against CU can be attributed to high polymer/ceramic ratio [20], whereas the statistically higher mass loss of the antagonist against MII, irrespective of the media, verifies that MII is a hard material with a hardness of 6.2 GPa [34]. When a hard surface comes in contact, wear would be certain. Here it is noteworthy that wear behavior is comprised of complex multifactorial variables involving microstructure, porosity, particle size, filler contents, the surface hardness of both material and the antagonist and the environment where wear occurs [7,35]. All the tested CAD/CAM materials demonstrated a statistically significant increase in surface roughness following chewing simulation (*p* < 0.05).

Our study appraised the performance of the materials after 7 days in an acidic drink, as many reports in dental literature evaluated similar time intervals [6,8,26,32]. Yet, the plausible findings of this study should be interpreted with caution as other factors and the oral environment must be considered. The actual results could vary depending on routine intra-oral activities. More studies are strongly recommended to yield a better understanding of different CAD/CAM material behaviors.

## 5. Conclusions

The hardness of the tested CAD/CAM materials is significantly affected when the materials are exposed to a low pH acidic environment for 7 days. The immersion process followed by simulated chewing demonstrated a deleterious effect on the roughness of the materials. The wear of antagonists after 120,000 chewing cycles were higher against MII material followed by VE. The hardness of Cerasmart was not influenced by the acidic environment and caused least wear of the antagonist. Cerasmart material could be recommended in clinical situations requiring less wear of the antagonist. However, other mechanical and surface properties should be considered before its clinical applications. Future studies should be directed towards evaluating the color stability and fatigue properties of the tested CAD/CAM materials in an acidic environment.

## Figures and Tables

**Table 1:** Three-way ANOVA for the effect of independent factors and their interaction on microhardness.

Source	Type III Sum of Squares	df	Mean Square	F	*p*
Corrected Model	6,796,798.40	19	357,726.23	2173.01	<0.001 *
Intercept	9,374,839.70	1	9,374,839.70	56,947.63	<0.001 *
Materials	6,726,658.12	4	1,681,664.53	10,215.30	<0.001 *
Storage environment	9645.21	1	9645.21	58.59	<0.001 *
Treatment time	6770.98	1	6770.98	41.13	<0.001 *
Materials × Storage environment	11,669.75	4	2917.43	17.72	<0.001 *
Materials × Treatment time	16,106.17	4	4026.54	24.45	<0.001 *
Storage environment × Treatment time	11,782.66	1	11,782.66	71.57	<0.001 *
Materials× Storage environment × Treatment time	14,165.49	4	3541.37	21.51	<0.001 *
Error	29,631.97	180	164.62		
Total	16,201,270.09	200			
Corrected Total	6,826,430.38	199			

* Statistically Significant (*p* < 0.05).

**Table 2:** Three-way ANOVA for the effect of independent factors and their interaction on surface roughness.

Source	Type III Sum of Squares	df	Mean Square	F	*p*
Corrected Model	30.97	29	1.06	38.20	<0.001 *
Intercept	42.26	1	42.26	1511.34	<0.001 *
Materials	1.29	4	0.32	11.58	<0.001 *
Storage environment	28.16	2	14.08	503.56	<0.001 *
Treatment time	0.00	1	0.00	0.04	0.828
Materials × Storage environment	1.02	8	0.12	4.60	<0.001 *
Materials × Treatment time	0.30	4	0.07	2.69	0.031 *
Storage environment × Treatment time	0.02	2	0.01	0.43	0.646
Materials × Storage environment × Treatment time	0.16	8	0.02	0.73	0.661
Error	7.55	270	0.02		
Total	80.78	300			
Corrected Total	38.52	299			

* Statistically Significant (*p* < 0.05).

**Table 3:** Percent mass loss of the antagonist against the tested CAD/CAM materials.

Material	Storage Media	Baseline Mass	Final Mass	Mass Loss (%)	*Significance*
CU	AS	4.0479	4.0471	0.019	*p* > 0.05
	Cola	4.0490	4.0481	0.022	*p* > 0.05
VE	AS	4.0581	4.0572	0.022	*p* > 0.05
	Cola	4.0674	4.0664	0.024	*p* > 0.05
LU	AS	4.1936	4.1927	0.018	*p* > 0.05
	Cola	4.0347	4.0339	0.019	*p* > 0.05
CS	AS	4.1457	4.1455	0.004	*p* > 0.05
	Cola	4.1322	4.1319	0.007	*p* > 0.05
MII	AS	4.0595	4.0570	1.231	*p* < 0.05 *
	Cola	4.1454	4.0708	1.801	*p* < 0.05 *

* Statistically significant (*p* < 0.05).

## Data Availability

Data sharing is not applicable to this article.
